# Potentially clinically significant drug-drug interactions in older patients admitted to the hospital: A cross-sectional study

**DOI:** 10.3389/fphar.2023.1088900

**Published:** 2023-02-02

**Authors:** Zuzana Očovská, Martina Maříková, Jiří Vlček

**Affiliations:** ^1^ Department of Social and Clinical Pharmacy, Faculty of Pharmacy in Hradec Králové, Charles University, Hradec Králové, Czech Republic; ^2^ Department of Clinical Pharmacy, Hospital Pharmacy, University Hospital Hradec Králové, Hradec Králové, Czech Republic

**Keywords:** hospitalization, Czech Republic, adverse drug event, drug drug interaction, older patients

## Abstract

**Background:** An international consensus list of potentially clinically significant drug-drug interactions (DDIs) in older people has been recently validated. Our objective was to describe the prevalence and characteristics of drug combinations potentially causing clinically significant DDIs identified in the medication history of older patients admitted to the hospital and the prevalence and characteristics of manifest DDIs–DDIs involved in adverse drug events present at hospital admission, DDIs that contributed to ADE-related hospital admissions, and DDIs involved in drug-related laboratory deviations.

**Methods:** The data were obtained from our previous study that examined the drug-relatedness of hospital admissions to University Hospital Hradec Králové *via* the department of emergency medicine in the Czech Republic. Patients ≥ 65 years old were included. Drug combinations potentially causing clinically significant DDIs were identified using the international consensus list of potentially clinically significant DDIs in older people.

**Results:** Of the 812 older patients admitted to the hospital, 46% were exposed to drug combinations potentially causing clinically significant DDIs. A combination of medications that affect potassium concentrations accounted for 47% of all drug combinations potentially causing clinically significant DDIs. In 27 cases, potentially clinically significant DDIs were associated with drug-related hospital admissions. In 4 cases, potentially clinically significant DDIs were associated with ADEs that were present at admissions. In 4 cases, the potentially clinically significant DDIs were associated with laboratory deviations. Manifest DDIs that contributed to drug-related hospital admissions most frequently involved antithrombotic agents and central nervous system depressants.

**Conclusion:** The results confirm the findings from the European OPERAM trial, which found that drug combinations potentially causing clinically significant DDIs are very common in older patients. Manifest DDIs were present in 4.3% of older patients admitted to the hospital. In 3.3%, manifest DDIs contributed to drug-related hospital admissions. The difference in the rates of potential and manifest DDIs suggests that if a computerized decision support system is used for alerting potentially clinically significant DDIs in older patients, it needs to be contextualized (e.g., take concomitant medications, doses of medications, laboratory values, and patients’ comorbidities into account).

## Introduction

Multimorbidity is highly prevalent in our aging societies, and it often leads to the use of multiple medications in older patients. Following recommendations for prescription in clinical guidelines will result in several potentially serious drug-drug interactions (DDIs) (Dumbreck et al., 2015). Drug regimens are increasingly complex and potentially harmful, and people with polypharmacy need regular review and prescribing optimization (Guthrie et al., 2015). Polypharmacy might represent either appropriate polypharmacy or problematic polypharmacy. Appropriate polypharmacy is the concurrent use of multiple medications by one individual when medication use has been optimized and when the medications are prescribed according to the best evidence. Problematic polypharmacy is the concurrent use of multiple medications by one individual when medications are prescribed inappropriately or when the intended benefit of the medication is not realized (McCarthy et al., 2019).

Older patients are at higher risk of adverse drug events (ADEs) from DDIs due to age-related changes in pharmacokinetics and pharmacodynamics and a higher number of comorbidities and medications. Several population-based studies have reported significant harm associated with DDIs in older patients (Hines and Murphy, 2011).

Our findings suggest that more than two-thirds of patients admitted to the hospital *via* the emergency department have at least one potential DDI in their medication history (Očovská et al., 2021). Fortunately, only a few of these combinations potentially causing DDIs are contraindicated or require drug dosage adjustments (Očovská et al., 2022b). The most common management strategies suggested by DDI databases all concern monitoring (Očovská et al., 2022b). Moreover, for many potential DDIs, there is a theoretical potential for an adverse interaction to occur based on the known pharmacological properties of the administered drugs, but no clinically relevant adverse effect (Pirmohamed, 2010). As a consequence, potential DDIs far outnumber actual DDIs (Pirmohamed, 2010; Magro et al., 2012; Očovská et al., 2021). Concerns about DDIs for which no clinical outcome evidence exists might lead to the underuse of safe and effective medications (Bykov and Gagne, 2017). It would mean that the evidence-based benefits of the medications are ignored in the face of a theoretical potential for harm (Pirmohamed, 2010). Just as harm associated with DDIs is usually avoidable, suboptimal patient outcomes due to the underuse of evidence-based medications are also usually avoidable (Bykov and Gagne, 2017). The omission of recommended drug therapy is associated with negative health outcomes, including reduced quality of life and a greater risk of hospitalizations or death. In comparison to younger populations, older patients are more likely to suffer adverse consequences from both action and inaction (Sloane and Niznik, 2022).

Tukukino et al. have shown that interaction alerts are of questionable value as indicators of problematic prescribing. Most alerts are either already being addressed or are not relevant in the clinical setting. The identification of DDIs using DDI databases thus results in many DDIs which might not be clinically significant (Tukukino et al., 2022). Recently, an international consensus list of potentially clinically significant DDIs in older people has been validated (Anrys et al., 2021). However, the association of DDIs listed in the international consensus list with clinical manifestations has never been examined.

Therefore, our objective was not only to describe the prevalence and characteristics of potentially clinically significant DDIs recorded in medication history but also to describe the prevalence and characteristics of manifest/actual DDIs (DDIs associated with ADE-related hospital admissions, ADEs that were present at hospital admissions and laboratory deviations).

## Methods

This is a sub-study of our previous observational study, which has been described earlier (Očovská et al., 2022a). The study examined the drug-relatedness of hospital admissions to the University Hospital Hradec Králové *via* the department of emergency medicine in August–November 2018. The number of hospital admissions *via* the department of emergency medicine of the University Hospital Hradec Králové is approximately 450 per month. The exclusion criteria included visits to the department of emergency medicine without inpatient hospitalization, hospitalizations for diagnostic or elective surgical procedures for pre-existing conditions, hospitalizations with missing medical records, and hospitalizations taking less than 24 h. We have not applied any exclusion criteria related to the type of medical ward. Most of the patients were admitted to the departments of internal medicine (49%), surgery (26%), neurology (10%), pneumology (4%), anesthesiology, resuscitation and intensive medicine (3%), oncology and radiotherapy (3%), orthopedics (2%), infectious diseases (1%), and psychiatry (1%). In this sub-study, we analyzed only hospital admissions of older patients (≥ 65 years old).

The design of the original study was cross-sectional–we have examined each patient’s medical record only at one point in time (we have not followed the patients in time). The data collection was performed retrospectively during 2018–2021. Data were obtained from electronic medical records and entered into a Microsoft Access database. The collected data included demographic characteristics, medication history, medical history, presenting complaint, admission diagnosis, laboratory values, results of clinical investigations, documented ADRs and information on medication adherence. Medications stated in medication history were counted as active substances.

### Identification of potentially clinically significant DDIs

Potentially clinically significant DDIs were identified using the international consensus list of potentially clinically significant DDIs in older people (Anrys et al., 2021). Potential harms resulting from these DDIs were classified according to Zerah et al. (2021) into the following categories: serious cardiovascular adverse effects; serious neurological adverse effects; bleeding; deterioration of renal function and/or hyperkalemia (including severe myopathy and rhabdomyolysis, which may lead to acute renal failure); hematologic toxicity; and miscellaneous others.

Potentially clinically significant DDIs should be interpreted as drug combinations potentially causing clinically significant DDIs.

### Outcome measures

The prevalence of hospital admissions with a potentially clinically significant DDI was calculated as the number of hospital admissions with at least one potentially clinically significant DDI according to the international consensus list (Anrys et al., 2021) divided by the total number of hospital admissions of older patients.

The prevalence of hospital admissions with a manifest DDI was calculated as the number of hospital admissions with at least one DDI according to the international consensus list (Anrys et al., 2021) that was associated with laboratory deviation, ADE that was present at hospital admission, or drug-related hospital admissions divided by the total number of hospital admissions of older patients.

Manifest DDIs included potentially clinically significant DDIs with potential harms that corresponded with observed clinical manifestations of ADE or laboratory deviations. The clinical adjudication process of drug-related hospital admissions has already been described in detail in our previous study (Očovská et al., 2022a). Drug-related hospital admissions were identified using the OPERAM drug-related hospital admissions adjudication guide (Thevelin et al., 2018). The process of drug-related hospital admissions identification consisted of data abstraction, screening for potential ADEs causing or contributing to hospital admission, causality assessment (using modified WHO-UMC criteria) and assessment of contribution to hospital admission.

### Statistical analysis

Data were analyzed using Microsoft Excel and IBM SPSS Statistics version 28. Descriptive statistics was performed in Microsoft Excel and multiple logistic regression was performed in IBM SPSS Statistics. We considered a *p*-value less than 0.05 as statistically significant.

## Results

**FIGURE 1:**
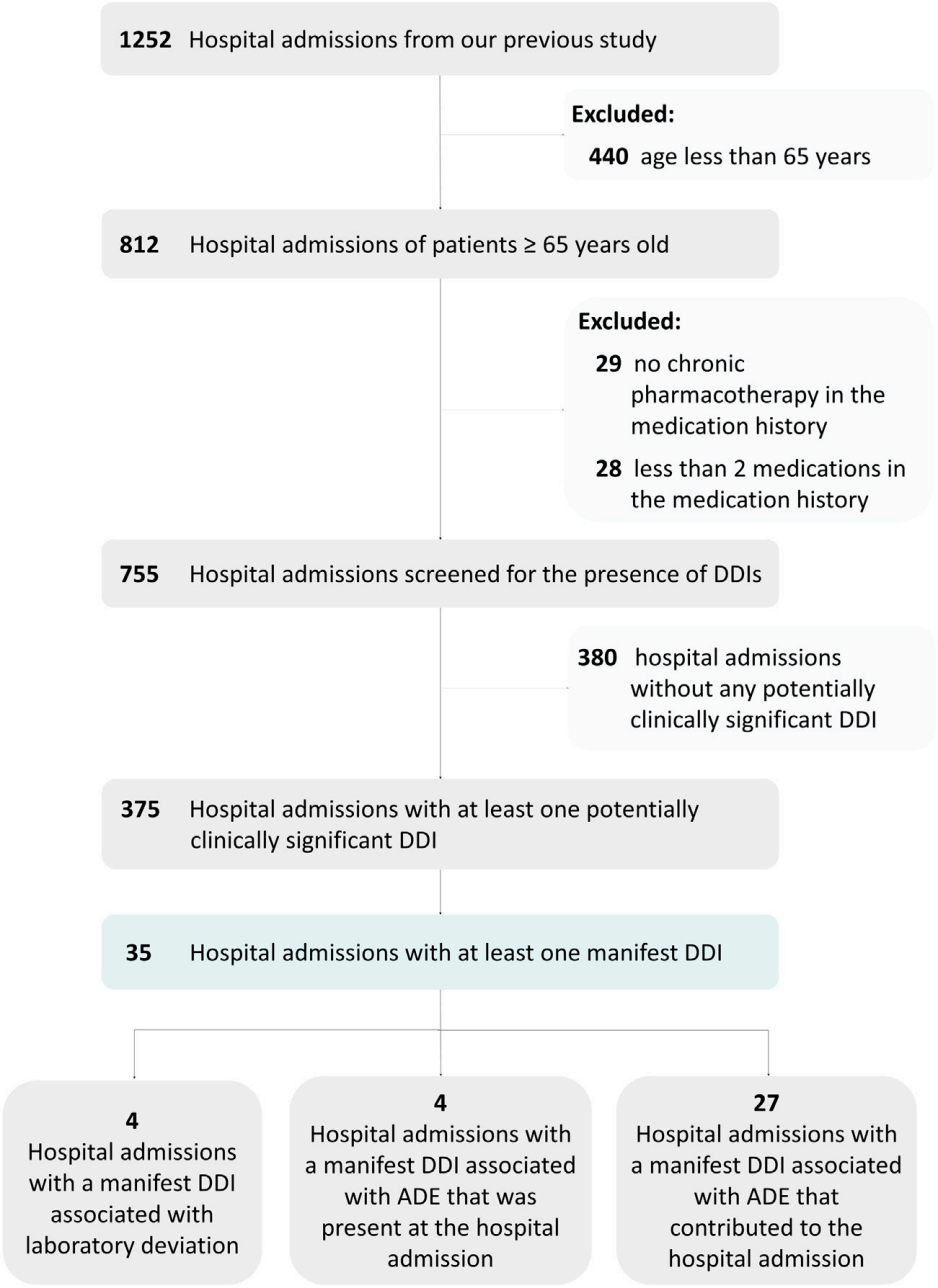
Flow chart showing the number of hospital admissions in each step.

Figure 1 shows the number of hospital admissions in each step of the study. Of 812 older patients admitted to the hospital, 375 patients (46%) had at least one drug combination potentially causing clinically significant DDI according to the international consensus list (Anrys et al., 2021) in the medication history. In 35 cases, potentially clinically significant DDIs were associated with clinical manifestations. The prevalence of hospital admissions with at least one manifest clinically significant DDI according to the international consensus list was 4.3%.

Descriptive characteristics of the study sample can be found in Supplementary Tables S1–S3. Polypharmacy (≥5 medications) was present in 597 (74%) patients and hyperpolypharmacy (≥ 10 medications) was present in 228 (28%) patients.

### Drug combinations potentially causing clinically significant DDIs

The most common medications involved in potentially clinically significant DDIs according to the international consensus list (Anrys et al., 2021) included furosemide, hydrochlorothiazide, fenoterol, amiodarone, acetylsalicylic acid, warfarin, amiloride, formoterol, spironolactone, ramipril, perindopril, potassium chloride, escitalopram, theophylline, atorvastatin, citalopram, tramadol, sertraline, ibuprofen, digoxin, diclofenac, and meloxicam. Supplementary Table S4 shows the most common potentially clinically significant DDIs according to the international consensus list (Anrys et al., 2021) that were listed in the medication history of older patients. Supplementary Table S5 shows medication classes involved in potentially clinically significant DDIs according to the international consensus list (Anrys et al., 2021). The most common medication classes involved in potentially clinically significant DDIs included Diuretics (C03), Drugs for obstructive airway diseases (R03), Antithrombotic agents (B01), Agents acting on the renin-angiotensin system (C09), Antiinflammatory and antirheumatic products (M01), Cardiac therapy (C01) and Psychoanaleptics (N06).

Potential harms of potentially clinically significant DDI according to the international consensus list (Anrys et al., 2021) included hypokalemia (*n* = 240), bleeding (*n* = 148), hyperkalemia (*n* = 139), CNS depression (*n* = 63), additive adverse effects on renal function (*n* = 52), hyponatremia (*n* = 45), myopathy (*n* = 42), digoxin toxicity (*n* = 26), serotonin syndrome (*n* = 24), bradycardia (*n* = 7), and anticholinergic effects (*n* = 6). Table 1 shows the overview of potentially clinically significant DDIs categorized to potential harms according to Zerah et al. (2021) and Table 2 shows the proportion of patients with the corresponding potential harm of potentially clinically significant DDIs according to Zerah et al., 2021. Potentially clinically significant DDIs involving drugs that affect potassium concentrations accounted for 47% of all potentially clinically significant DDIs according to the international consensus list (Anrys et al., 2021).

**TABLE 1:** The number of drug combinations potentially causing clinically significant DDIs with corresponding potential harm category according to Zerah et al., 2021.

Potential harm category		N of DDIs	% of DDIs
**Serious cardiovascular adverse effect**		**273**	**34.1**
hypokalemia		240	30.0
digoxin toxicity		26	3.3
bradycardia		7	0.9
**Deterioration of renal function or hyperkalemia**		**233**	**29.1**
hyperkalemia		139	17.4
additive adverse effects on renal function, antagonist effects on blood pressure		33	4.1
myopathy		42	5.3
deterioration of renal function, hyperkalemia, altered blood pressure control		19	2.4
**Bleeding**		**156**	**19.5**
bleeding		148	18.5
gastrointestinal ulceration or bleeding		8	1.0
**Serious neurologic adverse effects**		**93**	**11.6**
excessive sedation and prolonged hypnotic effects		6	0.8
increased risk of falls and fractures, impaired cognition		57	7.1
serotonin syndrome		24	3.0
anticholinergic effects including cognitive decline		6	0.8
**Others**		**45**	**5.6**
hyponatremia		45	5.6
Total		**800**	**100**

DDI: Drug-drug interaction.

Note: These drug-drug interactions should be interpreted as drug combinations potentially causing clinically significant DDIs, according to the international consensus list (Anrys et al., 2021).

**TABLE 2:** The proportion of patients with drug combinations potentially causing clinically significant DDIs with the corresponding potential harm category according to Zerah et al., 2021.

Potential harm category	N of patients	% of patients
Deterioration of renal function or hyperkalemia	184	23
Serious cardiovascular adverse effect	146	18
Bleeding	116	14
Serious neurologic adverse effects	72	9
Hyponatremia	42	5
Any harm category	375	46

n = 812 (100%).

Note: These drug-drug interactions should be interpreted as drug combinations potentially causing clinically significant DDIs, according to the international consensus list (Anrys et al., 2021).

184 (23%) patients had at least one potentially clinically significant DDI related to the deterioration of renal function or hyperkalemia. 146 (18%) patients had at least one potentially clinically significant DDI related to serious cardiovascular adverse effects. 116 (14%) patients had at least one potentially clinically significant DDI related to bleeding. 72 (9%) patients had at least one potentially clinically significant DDI related to serious neurologic adverse effects. 42 (5%) patients had at least one potentially clinically significant DDI related to hyponatremia.

### Manifest clinically significant DDIs

Table 3 shows the overview of manifest DDIs that were associated with drug-related hospital admissions. Manifest DDIs were involved in 27 drug-related hospital admissions. The most common clinical presentation of manifest DDIs was bleeding (especially gastrointestinal bleeding). Medication classes most frequently involved in manifest DDIs included antithrombotics (antiplatelets, anticoagulants) and CNS depressants.

**TABLE 3:** List of manifest DDIs that were associated with drug-related hospital admissions (n = 27).

Actual harm category	Manifest drug-drug interaction
Bleeding	apixaban + ASA
	ASA + warfarin + clopidogrel + escitalopram
	ASA + clopidogrel + rivaroxaban
	ASA + warfarin
	ASA + nimesulide
	ASA + warfarin + sertraline
	ASA + rivaroxaban
	NSAID + warfarin
	clopidogrel + warfarin
	ASA + ibuprofen
	ASA + diclofenac
	ASA + dabigatran etexilate + meloxicam
	clopidogrel + warfarin
	ibuprofen + rivaroxaban
	diclofenac + prednisone
	ASA + warfarin
	dabigatran etexilate + meloxicam
	clopidogrel + warfarin + ASA
	ASA + warfarin
CNS depression	pregabalin + tramadol + zolpidem
	baclofen + pregabalin + tramadol
	buprenorphine + gabapentin + trazodone
	dosulepin + tapentadol + tramadol + trazodone + pregabalin
	fentanyl + gabapentin + haloperidol + morphine
Hyperkalemia	perindopril + potassium chloride + spironolactone
	amiloride + telmisartan
	perindopril + spironolactone

ASA: acetylsalicylic acid, CNS: central nervous system, NSAID: non-steroidal anti-inflammatory drug.

Table 4 shows the lists of manifest DDIs that were associated with ADEs that were present at hospital admission but did not contribute to drug-related hospital admission (*n* = 4) and DDIs that were associated with drug-related laboratory deviations (*n* = 4). Medications with hyperkalemic effects–spironolactone, amiloride, angiotensin-converting enzyme (ACE) inhibitors, and angiotensin II receptor blockers (ARBs) were involved in DDIs that were associated with laboratory deviations (hyperkalemia).

**TABLE 4:** List of other manifest DDIs (*n* = 8).

Manifest drug-drug interaction	Adverse drug event or laboratory deviation
*DDIs involved in adverse drug events that were present at hospital admission (n = 4)*	
ASA + rivaroxaban	gastroduodenal hemorrhage
gabapentin + trazodone + zolpidem	abnormal dreams
olanzapine + solifenacin	constipation
clonazepam + quetiapine + trazodone	CNS depression
*DDIs involved in drug-related laboratory deviations (n = 4)*	
spironolactone + telmisartan	hyperkalemia 7.5 mmol/L
perindopril + spironolactone	hyperkalemia 5.4 mmol/L
amiloride + perindopril + spironolactone	hyperkalemia 9.0 mmol/L
furosemide + hydrochlorothiazide	hypokalemia 2.9 mmol/L

ASA: acetylsalicylic acid, CNS: central nervous system, DDI: drug-drug interaction.

In addition, there were ten additional cases with manifest DDIs that were not included in the international consensus list of potentially clinically significant DDIs in older people (Anrys et al., 2021).

## Discussion

### Prevalence of drug combinations potentially causing clinically significant DDIs

We have found that almost half of the patients (46%) admitted to the hospital were exposed to potentially clinically significant DDIs according to the international consensus list (Anrys et al., 2021). This prevalence is lower than the prevalence of 54% found in the OPERAM trial (Zerah et al., 2021). However, if we restricted our sample only to similar patients as in the OPERAM trial (≥70 years, with ≥ 3 chronic conditions) and polypharmacy (≥ 5), we would find a slightly higher prevalence of potentially clinically significant DDIs (58%) (303/523).

If we looked at the prevalence of any potential DDIs (not only potentially clinically significant DDIs in older people), the prevalence of potential DDIs would be 85%. Only in 63 cases with at least two medications in the medication history, there was no DDI identified either by Lexicomp, Micromedex, or Stockley drug interaction databases.

Therefore, limiting the identification of DDIs to those listed in the international consensus list of potentially clinically significant in older people has almost halved the prevalence of potential DDIs.

### Medications involved in drug combinations potentially causing clinically significant DDIs

In the OPERAM trial, 80% of all potentially clinically significant DDIs involved drugs that reduce potassium (diuretics, inhaled beta2-agonists, systemic corticosteroids), centrally acting drugs (psychotropics, antidepressants, opioids, antiepileptics), potassium-sparing drugs (ACE inhibitors, ARBs, spironolactone) and antithrombotics (Zerah et al., 2021).

In our study, DDIs most frequently included a combination of medications that reduce potassium (DDI No. 65), a combination of medications that increase potassium (DDI No. 21 + 22 + 23), a combination of an oral anticoagulant with an antiplatelet drug (DDI No. 12), and concomitant use of ≥ 3 centrally-acting drugs (DDI 36). In 70 cases, both DDIs involving drugs that reduce potassium and DDIs involving drugs that increase potassium were present at the same time, which highlights the need for contextualization of DDIs alerts.

### The most common potential harm of drug combinations potentially causing clinically significant DDIs

Hypokalemia represented the most common potential harm of potentially clinically significant DDIs according to the international consensus list (Anrys et al., 2021). Manifestations of hypokalemia include muscle weakness, constipation, cardiac arrhythmias, kidney abnormalities, and glucose intolerance. Although hypokalemia represented the most common type of potential harm of potentially clinically significant DDIs in our study, we have not detected any ADEs associated with hypokalemia. Thiazide diuretics were often prescribed in fixed combination with ACE inhibitors, ARBs, or amiloride. The risk was further minimized by using lower doses of thiazide diuretics. Spironolactone and ACE inhibitors were often prescribed in patients with heart failure (heart failure represented the most common admission diagnosis in our study). In addition, medications frequently implicated in potential DDIs associated with hypokalemia included inhaled beta 2 agonists, which do not have a high potential to cause hypokalemia.

Due to the hospital setting of our study, we could only identify cases of hypokalemia with severe types of manifestations (e.g., arrhythmias) as we did not prospectively look for the patient’s reported symptoms (e.g., muscle weakness) outside of the hospital setting. There were very few cases of hypokalemia in our study, and they were mostly related to vomiting, diarrhea, or excessive alcohol use.

### Prevalence of manifest DDIs

In our study, the prevalence of hospital admissions with at least one manifest DDIs according to the international consensus of potentially clinically significant DDIs was 4.3%. This prevalence is higher compared to the median DDI prevalence of 1.1% from the latest systematic review (Dechanont et al., 2014).

However, there are also a few studies with a higher prevalence of DDI-related hospital admissions. In a study from Australia, DDIs were potentially involved in 8.1% of all hospital admissions and 43% of ADR-related admissions (Parameswaran Nair et al., 2017). In a study from Italy, an actual DDI was found in 5.5% of emergency department admissions (Marino et al., 2016). A study from the USA reported that DDIs were the cause of 57% of ADR-related admissions and 4.3% of all hospital admissions. (Rivkin, 2007). The latest systematic review indicated that in ADR patients, the median DDI prevalence rate for hospital admissions is 22.2%. (Dechanont et al., 2014). A recent study (Osanlou et al., 2022) found that 29.4% of ADRs are possibly or probably caused by DDIs.

The prevalence of hospital admissions associated with DDIs ranges from 0% (Hohl et al., 2001) to 18% (De Paepe et al., 2013). The prevalence of hospital admissions related to manifest DDIs is influenced by various factors such as characteristics of the studied population (e.g., age, number of comorbidities, number of medications), the definition of manifest DDI, the method used to identify DDIs, the method of causality assessment, the selected causality threshold, the assessment of contribution to hospital admission, and the emergence of new evidence of ADEs associated with DDIs.

### Factors that influence the manifestation of potential DDIs

**FIGURE 2:**
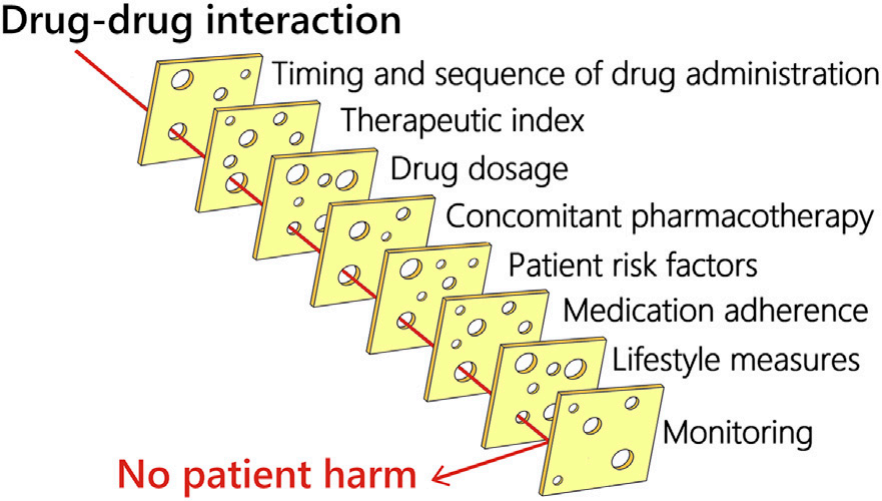
Factors that might influence whether potential DDI will lead to patient harm.

Several factors influence the manifestation of potential DDIs. These factors can be related to the medication (e.g., therapeutic index, drug dosage or duration of treatment, other concomitant pharmacotherapy), patient characteristics (e.g., genetic polymorphism, the status of eliminating organs and comorbidities), drug administration (route, sequence, and correct way of drug administration), and patient behavior (medication adherence, self-monitoring, lifestyle measures). Lifestyle measures such as consumption of certain foods and beverages, hydration, smoking, and alcohol consumption also represent a source of variability. Last but not least, healthcare professionals minimize the risk of DDIs by monitoring (e.g., monitoring drug levels, potassium levels, kidney functions, blood pressure, heart rate, QTc interval, and symptoms of ADEs). Figure 2 shows the various factors that might influence whether potential DDI will lead to patient harm.

### DDIs not included in the international consensus list

DDIs that were not listed in the international consensus list of potentially clinically significant DDIs in older patients but were associated with drug-related hospital admissions in our study included the combinations of selective serotonin reuptake inhibitors (SSRIs) with antithrombotic agents (both anticoagulant and antiplatelets), the combination of two antiplatelet agents (acetylsalicylic acid and clopidogrel), the combinations of beta-blockers with amiodarone or digoxin and the combinations of several medications with hypotensive effect.

Considering that bleeding represents the most common clinical manifestation of DDI-related hospital admissions, additional DDIs related to increased risk of bleeding should be considered during the development of an updated list of potentially clinically significant DDI in older adults. Gastrointestinal hemorrhage represented the most common ADE also in our previous study focused on older patients admitted to the geriatric ward (Maříková et al., 2021). A combination of two antiplatelet agents was frequently implicated in serious ADRs associated with DDIs identified *via* a spontaneous reporting database from Italy (Magro et al., 2020). In a pharmacovigilance study from China (Jiang et al., 2022), acetylsalicylic acid represented the most common medication implicated in ADRs caused by actual DDIs. The inclusion of a combination of antidepressants belonging to the SSRI and SNRI class with antithrombotics should also be considered. In the meta-analysis of 32 non-randomized studies (Nochaiwong et al., 2022), serotonin reuptake inhibitor (SRI) antidepressants among patients treated with antithrombotic therapy (either anticoagulant or antiplatelet) were associated with a higher risk of bleeding complications. The combination of vitamin K antagonist with SSRI/SNRI is also included in the Ghent Older People’s Prescriptions Community Pharmacy Screening list of DDIs especially relevant in older people (Foubert et al.,2021).

In the current version of the international consensus list of potentially significant DDIs, most DDIs affecting CNS were only included when patients were taking three or more centrally-acting drugs. Nevertheless, the list could also include the combination of opioids with benzodiazepines and the combination of opioids with gabapentinoids as recommended by AGS Beers criteria (AGS, 2019). In addition, the combination of skeletal muscle relaxants with opioids and benzodiazepines is not included in the international consensus list. Concomitant use of specific muscle relaxants (e.g., baclofen), benzodiazepines, and gabapentinoids might increase the risk of opioid overdose (Li et al., 2020; Khan et al., 2021; Khan et al., 2022) and the risk of injuries (Leonard et al., 2020).

Moreover, compared to younger patients, older patients do not require too tight blood pressure and glycemic control. Fortunately, due to the development of new oral antidiabetics, the combinations of antidiabetics with the risk of hypoglycemia are not common in clinical practice. However, the combination of oral antidiabetics with a risk of hypoglycemia (sulphonylureas) or insulin with beta-blockers might result in masking the first symptoms of hypoglycemia (tachycardia, tremor). On the other hand, the combinations of several medications with hypotensive effects are common in clinical practice. Hypotension caused by multiple blood pressure-lowering agents was reported in a study from Australia (Parameswaran Nair et al., 2017). Conversely, medications that antagonize the effect of ACE inhibitors/ARBs or diuretics (e.g., NSAIDs) might contribute to heart failure exacerbations (Page et al., 2016; Swart et al., 2020).

### Risk minimization of adverse drug events

Since gastrointestinal bleeding represented the most common ADE associated with manifest DDIs in our study, DDIs that increase the risk of bleeding or gastrointestinal ulceration deserve attention. Risk minimization measures should target inappropriate prescriptions of antiplatelet agents and NSAIDs. Low-dose acetylsalicylic acid use is not recommended for the primary prevention of cardiovascular disease. Since the risk of major bleeding from acetylsalicylic acid increases in older patients, initiation of low-dose acetylsalicylic acid for primary prevention of cardiovascular disease should be avoided and deprescribing should be considered in older patients already taking low-dose acetylsalicylic acid for primary prevention. (2022 AGS Annual Scientific Meeting). For patients with atrial fibrillation on anticoagulation who underwent percutaneous coronary intervention, the use of direct oral anticoagulants is preferred over a vitamin K antagonist when appropriate. Clinical decision-making regarding the duration of antiplatelet therapy should be based on a balanced assessment of three competing risks: cardioembolic stroke, coronary ischemic events, and bleeding. In patients with a low risk of thrombotic events or a high risk of bleeding, early omission of aspirin therapy and treatment with a direct oral anticoagulant plus clopidogrel is entirely warranted (Mehta, 2019). In general, the use of triple therapy (dual antiplatelet therapy plus anticoagulation) is not recommended for most patients due to an increased risk of bleeding. If triple therapy is needed, a short duration (e.g., no more than 30 days) is recommended (Kumbhani et al., 2021). A screening tool for cardiovascular pharmacotherapy in geriatric patients (RASP_CARDIO list) states that triple therapy (dual antiplatelet therapy and one anticoagulant) longer than 1 month after a percutaneous coronary intervention is potentially inappropriate. Treatment duration is preferably limited to 1 week (with mostly stepping down to dual antithrombotic therapy upon discharge from the hospital) (De Schutter et al., 2022). For patients taking two antithrombotic agents, starting or continuing a proton pump inhibitor and avoiding NSAIDs should be employed to reduce gastrointestinal bleeding risk. However, while proton pump inhibitors reduce the risk of upper gastrointestinal bleeding, the risk of lower gastrointestinal bleeding is not reduced. In addition, proton pump inhibitors might be implicated in ADRs that lead to hospital admissions, e.g., due to *C. difficile* enterocolitis (Osanlou et al., 2022).

Risk minimization of CNS adverse events should focus on off-label prescription of psychotropic drugs–particularly the use of benzodiazepines and antipsychotics should be avoided except in approved evidence-based indications. Non-pharmacologic treatment of insomnia and depression should be promoted. Deprescribing opioids and gabapentinoids might be complicated by the lack of safe and effective alternatives for pain control in older adults. Paracetamol dosages should be checked and possibly increased (up to 1,000 mg) in patients with inadequate pain management. In our study, paracetamol doses of 325–650 mg (paracetamol in fixed combinations with tramadol) or 500 mg were often used. Perhaps, the use of metamizole (dipyrone) for chronic pain could be reevaluated in some countries in light of the high burden of ADRs associated with NSAIDs, opioids, and gabapentinoids. Start low and go slow dosing of many CNS medications is recommended in older patients. Furthermore, CYP2D6 activity affected by genotype and drug exposure (including DDIs) might influence the CNS’s vulnerability to ADRs (Just et al., 2021). In the future, the use of pharmacogenetics might increase drug safety by optimizing individual drug treatment (Evans and Relling, 2004).

Risk minimization of hyperkalemia should focus on slow titration of ACE inhibitors/ARBs and spironolactone during the initiation of the treatment of heart failure (start low and go slow approach). In addition, kidney function and potassium levels should be closely monitored, and medication reconciliation should be in place to avoid situations in which patients are being discharged with potassium chloride once hypokalemia has resolved. A recent study from the United States found a high incidence of loop diuretic-potassium supplementation prescribing cascade, with up to one-third of patients continuing to receive potassium supplementation despite loop diuretic discontinuation (Wang et al., 2022).

### Future studies

First of all, future studies on DDIs should assess the evidence of clinical outcomes of DDIs. An absence of evidence about whether a drug-drug interaction affects clinical outcomes not only contributes to DDI alert overload but can also result in suboptimal patient outcomes due to the underutilization of safe and effective medications (Bykov and Gagne, 2017). Bykov and Gagne have highlighted the urgent need for more and better pharmacoepidemiologic studies to understand the clinical impact, or lack thereof, of pharmacologically demonstrated DDIs (Bykov and Gagne, 2017). The evidence of clinical outcomes would benefit from more studies with a self-controlled design (particularly self-controlled case series) which is suited for the evaluation of transient effects of drug-drug interactions and controls for confounders that are stable over the observational period (Bykov et al., 2019).

Furthermore, studies should also focus on higher-order interactions. Drug-drug-drug signal detection using pharmacoepidemiologic screening of health insurance data could have broad applicability across drug classes and databases (Acton et al., 2022).

Most importantly, there is a need to contextualize DDI alerts so that computerized systems alert those DDIs that are relevant to the patient’s clinical situation. Clinical decision support systems tools need to be contextualized by taking clinical, user, and institutional factors into consideration (Chou et al., 2021). Warnings for DDIs are frequently overridden because they are often irrelevant for specific patients. Alerting systems for DDIs should incorporate patients’ comorbidities (e.g., chronic kidney disease, history of gastrointestinal bleeding), laboratory results (e.g., potassium, blood pressure, QTc values), drug dosages, duration and route of administration, and most importantly concomitant pharmacotherapy (particularly the presence of various DDIs affecting potassium). Concomitant pharmacotherapy can either reduce the clinical relevancy of a DDI by antagonistic effect (simultaneous presence of DDIs that reduce and increase serum potassium level) or further increase the clinical relevancy by synergistic effect (high-order drug interactions involving antithrombotic agents, antiplatelet agents, NSAIDs, and serotonin reuptake inhibitor antidepressants). A problematic issue related to DDI databases is generalizing evidence to members of a drug class and not distinguishing the clinical relevancy between different members of the same drug class. For example, metamizole (dipyrone) generates theoretical DDIs that affect blood pressure and kidney functions due to being listed among other NSAIDs. Recently Wasylewicz et al. have shown that contextualized DDI management can considerably decrease the number of irrelevant DDI alerts and thereby increase the time available to interpret relevant DDI alerts (Wasylewicz et al., 2022). Although it may be difficult to operationalize certain factors to reduce unnecessary alerts, these factors can provide useful information for clinicians to decide whether to override an alert (Reese et al., 2022).

### Strengths

The key strength of this study is the assessment of clinical manifestations associated with potentially clinically significant DDIs–laboratory deviations, ADEs that were present at admission, and drug-related hospital admissions. The second strength is the use of electronic health records as a data source. Compared to administrative claims data or spontaneous reporting systems, electronic health records are more likely to capture ADEs associated with DDIs. Electronic health records include presenting complaints, hospital discharge summaries, patient history, results of investigations, and various free text notes which are not available in other data sources. The third strength of this study is the use of the OPERAM drug-related hospital admissions adjudication guide for the identification of drug-related hospital admissions. This standardized guide provides comprehensive information on the definition, screening, and adjudication of drug-related hospital admissions (including ADE causality assessment and assessment of ADE contribution to hospital admission).

In addition, the study was not limited to specific hospital wards or a specific subgroup of older adults, thereby increasing its generalizability. However, since the study was focused on older adults acutely admitted to the hospital *via* the department of emergency medicine, we do not have any information on ADEs that did not result in hospital admissions of older patients. Although the study was single-centered, we have identified almost the same prevalence and characteristics of potentially clinically significant DDIs as the four medical centers from the OPERAM trial (Bern, Brussels, Cork, Utrecht). This study, therefore, contributes to existing knowledge on DDIs in older adults by providing information on the prevalence and characteristics of potentially clinically significant DDIs (medications involved in DDIs, potential harms of DDIs) from a different country.

The study provides additional evidence concerning actual clinical manifestations associated with potentially clinically significant DDIs in older adults. This is the first time that the international consensus list of potentially clinically significant DDIs in older adults has been used to explore drug-related hospital admissions. The information on manifest DDIs has extended our knowledge of the clinical relevance of potentially clinically significant DDIs in older adults. The identified difference between the prevalence of potentially clinically significant DDIs and the prevalence of manifest DDIs adds to a growing body of literature on the need to contextualize DDI alerts.

### Limitations

The main limitation of this study is the cross-sectional design. Since we were not able to follow patients in time, we did not have precise information on the time of initiation of each medication. In a prospective cohort study from Ireland, the authors were able to classify identified DDIs as chronic and acute (Hughes et al., 2021). Certain pharmacokinetic DDIs are only relevant when the object drug is initiated, discontinued, or dosage changes are made. Due to a lack of information on the duration of treatment, we were not able to assess the causality of amiodarone + warfarin DDI. Other DDIs were either pharmacodynamic or not associated with any relevant clinical manifestation.

The second limitation concerns the absence of patient interviews. Due to missing patient interviews, we do not have precise information on medication adherence and the use of over-the-counter medications and supplements. The imprecise information on NSAID use represents a major drawback of the study since gastrointestinal bleeding is the most frequent cause of drug-related hospital admissions. Although we have identified some cases of DDIs that involved the combination of NSAIDs with anticoagulants and antiplatelets, the magnitude of gastrointestinal bleeding associated with NSAIDs is likely greater. According to the systematic review, NSAIDs represent the most common drugs involved in hospital admissions associated DDIs (Dechanont et al., 2014). In addition, the adverse impact of DDIs on the quality of life remains unknown.

Moreover, fixed combinations consisting of two active ingredients were coded as two different active ingredients. The prevalence of hypokalemia is overestimated because the combination of hydrochlorothiazide and amiloride was also implicated in DDIs that potentially lead to hypokalemia.

## Conclusion

The results confirm the findings from the European OPERAM trial, which found that drug combinations potentially causing clinically significant DDIs are very common in older patients. Manifest DDIs were present in 4% of older patients admitted to the hospital. In 3%, manifest DDIs contributed to drug-related hospital admissions. The difference in the prevalence of potential and manifest DDIs suggests that if a computerized decision support system is used for alerting potentially clinically significant DDIs in older patients, it needs to be contextualized (e.g., take concomitant medications, doses of medications, laboratory values, and patients’ comorbidities into account).

## Manuscript contribution to the field

This is the first study that applied the International Consensus List of Potentially Clinically Significant Drug-Drug Interactions in Older People outside of the OPERAM trial. The results confirm the findings from the European OPERAM trial, which found that potentially clinically significant DDIs are very common in older patients. This study has identified potentially clinically significant drug-drug interactions that were missed in the consensus list (the combination of anticoagulants with SSRI antidepressants, the combination of two antiplatelet agents, and the combination of opioids with gabapentinoids). Therefore, this study could serve as an important guide for the development of the updated version of the international consensus list of potentially clinically significant drug-drug interactions in older people.

The strengths of this study include the assessment of clinical manifestations associated with drug-drug interaction in older patients (particularly drug-related hospital admissions) as well as laboratory deviations and adverse drug events that were present at hospital admission. The assessment of drug-related hospital admissions was performed using a standardized drug-related hospital admission adjudication guide developed during the European OPERAM trial.

The paper also proposed possible risk minimization measures for the most common ADEs associated with drug-drug interactions (bleeding, CNS depression, hyperkalemia), highlighted the factors that influence the manifestation of drug-drug interactions, and the importance of contextualization (e.g., taking concomitant medications, doses of medications, laboratory values, and patients’ comorbidities into account).

## Data Availability

The raw data supporting the conclusions of this article will be made available by the authors, without undue reservation.

## Abbreviations

ACE; Angiotensin-converting enzyme, ADE; Adverse drug event, ADR; Adverse drug reaction, ARB; Angiotensin II receptor blockers (AT1 receptor antagonists), ASA; Acetylsalicylic acid, CNS; Central nervous system, DDI; Drug-drug interaction, NSAID; Non-steroidal anti-inflammatory drug, OR; Odds ratio, SSRI; Selective serotonin reuptake inhibitors, SNRI; Serotonin and norepinephrine reuptake inhibitors, SRI; Serotonin reuptake inhibitors.
