# Supplementary material for: Potentially clinically significant drug-drug interactions in older patients admitted to the hospital: A cross-sectional study
Source: Front Pharmacol. 2023 Feb 2;14:1088900. doi: 10.3389/fphar.2023.1088900 (PMC9932507; doi:10.3389/fphar.2023.1088900)
Supplement: Supplementary file 1 [file Table1.pdf]

Supplementary Material

Supplement to: Očovská Z, Maříková M, Vlček J. Potentially clinically significant drug-drug interactions in older patients admitted to the hospital: A cross-sectional study. *Front. Pharmacol.* 2023;14:1088900. doi: 10.3389/fphar.2023.1088900

This supplementary material has been provided by the authors to give readers additional information about their work.

Contents

1 Sample characteristics 2

Supplementary Table S1: Demographic characteristics of the sample of older patients with at least two medications in the medication history 2

Supplementary Table S2: The most common baseline medications in a sample of older patients with at least two medications in the medication history 3

Supplementary Table S3: Comorbidities of the sample of older patients with at least two medications in the medication history 4

2 Potentially clinically significant drug-drug interaction in older adults 5

Supplementary Table S4: The most common potentially clinically significant DDIs according to Anrys et al. 2021 listed in the medication history of older patients.. 5

Supplementary Table S5: The most common combinations of medication classes that represented potentially clinically significant DDIs according to Anrys et al. 2021 and were listed in the medication history of older patients 6

3 Comorbidities associated with the presence of potentially clinically significant DDIs......... 7

Supplementary Figure S1: Comorbidities associated with the presence of potentially clinically significant DDIs and their possible relationships with indicated medication classes and potential harms resulting from potentially clinically significant DDI between these medication classes.. 7

1 Sample characteristics

Supplementary Table S1: Demographic characteristics of the sample of older patients with at least two medications in the medication history

Demographic characteristic	Value
**Age**	
median	79
IQR	72–86
**Sex**	
female – N (%)	386 (51)
male – N (%)	369 (49)
**Number of medications in the medication history**	
median	7
IQR	5–10
**Polypharmacy**	
≥ 5 medications – N (%)	597 (79)
≥ 10 medications – N (%)	228 (30)
**Charlson comorbidity index**	
median	5
IQR	4–7

n = 755 (100%) – the number of patients with at least two medications in the medication history
IQR: Interquartile range

Supplementary Table S2: The most common baseline medications in a sample of older patients with at least two medications in the medication history

Medication	N of patients	% of patients
acetylsalicylic acid (low-dose)	301	39.9
furosemide	240	31.8
atorvastatin	214	28.3
amlodipine	154	20.4
allopurinol	151	20.0
bisoprolol	151	20.0
hydrochlorothiazide	136	18.0
omeprazole	135	17.9
warfarin	122	16.2
perindopril	120	15.9
pantoprazole	119	15.8
levothyroxine sodium	119	15.8
metoprolol	117	15.5
ramipril	107	14.2
metformin	105	13.9
colecalciferol	101	13.4
amiloride	87	11.5
metamizole sodium	87	11.5
tamsulosin	83	11.0
spironolactone	76	10.1
ipratropium bromide	71	9.4
calcium carbonate	69	9.1
potassium chloride	62	8.2
tramadol	61	8.1
amiodarone	58	7.7
diosmin, combinations	55	7.3
fenoterol	54	7.2
telmisartan	51	6.8
indapamide	50	6.6
paracetamol	49	6.5
betaxolol	49	6.5
glimepiride	44	5.8
losartan	42	5.6
formoterol	41	5.4
zolpidem	41	5.4
clopidogrel	40	5.3
rosuvastatin	38	5.0
simvastatin	36	4.8
escitalopram	36	4.8
nadroparin	35	4.6
insulin glargine	34	4.5

n = 755 (100%) – the number of patients with at least two medications in the medication history

Supplementary Table S3: Comorbidities of the sample of older patients with at least two medications in the medication history

Comorbidity	N of patients	% of patients
Arterial hypertension	588	77.9
Dyslipidemia	331	43.8
Diabetes	286	37.9
Coronary artery disease	235	31.1
Valvular heart disease	198	26.2
Atrial fibrillation	195	25.8
Tumors	171	22.6
Vertebrogenic algic syndrome (chronic pain)	169	22.4
Heart failure	154	20.4
Post fracture	145	19.2
Chronic kidney disease	134	17.7
Benign prostatic hyperplasia	120	15.9
Osteoarthrosis	117	15.5
Hyperuricemia/gout	106	14.0
Post stroke	105	13.9
Post fall	103	13.6
Hypothyroidism	103	13.6
Chronic venous insufficiency	94	12.5
Anemia	91	12.1
Dementia	91	12.1
Peripheral artery disease	76	10.1
Venous thromboembolism	75	9.9
Chronic obstructive pulmonary disease	74	9.8
Osteoporosis	73	9.7
Peptic ulcer	63	8.3
Heart arrhythmia	62	8.2
Liver disease	62	8.2
Depression and/or anxiety	60	7.9
Gastroesophageal reflux disease	46	6.1
Asthma	44	5.8

n = 755 (100%) – the number of patients with at least two medications in the medication history

2 Potentially clinically significant drug-drug interaction in older adults

Supplementary Table S4: The most common potentially clinically significant DDIs according to Anrys et al. 2021 listed in the medication history of older patients

Potentially clinically significant DDIs in older people	N of patients	% of patients
furosemide + hydrochlorothiazide	35	4.6
fenoterol + furosemide	29	3.8
ASA + warfarin	28	3.7
amiodarone + atorvastatin	25	3.3
fenoterol + formoterol	22	2.9
amiodarone + warfarin	22	2.9
formoterol + furosemide	17	2.3
digoxin + furosemide	16	2.1
amiloride + ramipril	13	1.7
amiloride + perindopril	12	1.6
furosemide + sertraline	12	1.6
perindopril + potassium chloride	11	1.5
perindopril + spironolactone	11	1.5
potassium chloride + spironolactone	11	1.5
ramipril + spironolactone	11	1.5
fenoterol + theophylline	11	1.5
escitalopram + furosemide	10	1.3
fenoterol + hydrochlorothiazide	10	1.3
furosemide + theophylline	10	1.3
potassium chloride + ramipril	10	1.3
ASA + ibuprofen	9	1.2
apixaban + ASA	9	1.2
formoterol + hydrochlorothiazide	8	1.1
digoxin + hydrochlorothiazide	8	1.1
clopidogrel + warfarin	8	1.1
citalopram + furosemide	8	1.1
atorvastatin + verapamil	7	0.9
ASA + dabigatran etexilate	7	0.9
amlodipine + simvastatin	7	0.9
amiodarone + dabigatran etexilate	7	0.9
furosemide + chlortalidone	7	0.9
fenoterol + olodaterol	7	0.9
furosemide + prednisone	7	0.9
amiloride + telmisartan	7	0.9

n = 755 (100%) – the number of patients with at least two medications in the medication history
ASA: acetylsalicylic acid, DDI: Drug–drug interaction
Note: Central nervous system medications are not included in the table as there had to be a combination of three medications to be considered potentially clinically significant DDI (There were 57 additional potentially clinically significant DDIs that involved central nervous system medications)

Supplementary Table S5: The most common combinations of medication classes that represented potentially clinically significant DDIs according to Anrys et al. 2021 and were listed in the medication history of older patients

ATC code	ATC name	ATC code	ATC name	N
**C03**	Diuretics	**R03**	Drugs for obstructive airway diseases	109
**C03**	Diuretics	**C09**	Agents acting on the renin-angiotensin system	82
**R03**	Drugs for obstructive airway diseases	**R03**	Drugs for obstructive airway diseases	60
**B01**	Antithrombotic agents	**B01**	Antithrombotic agents	59
**C03**	Diuretics	**C03**	Diuretics	45
**C03**	Diuretics	**N06**	Psychoanaleptics	45
**B01**	Antithrombotic agents	**C01**	Cardiac therapy	38
**B01**	Antithrombotic agents	**M01**	Antiinflammatory and antirheumatic products	34
**C09**	Agents acting on the renin-angiotensin system	**M01**	Antiinflammatory and antirheumatic products	33
**C03**	Diuretics	**M01**	Antiinflammatory and antirheumatic products	30
**A12**	Mineral supplements	**C09**	Agents acting on the renin-angiotensin system	27
**C01**	Cardiac therapy	**C10**	Lipid modifying agents	27
**C01**	Cardiac therapy	**C03**	Diuretics	24
**N06**	Psychoanaleptics	**N02**	Analgesics	20
**C03**	Diuretics	**H02**	Corticosteroids for systemic use	17
**C08**	Calcium channel blockers	**C10**	Lipid modifying agents	15
**A12**	Mineral supplements	**C03**	Diuretics	13
**M01**	Antiinflammatory and antirheumatic products	**N06**	Psychoanaleptics	9
**H02**	Corticosteroids for systemic use	**M01**	Antiinflammatory and antirheumatic products	8

ATC: Anatomical Therapeutic Chemical
Note: While diuretics involved in potential hypokalemia included furosemide, hydrochlorothiazide, and indapamide, diuretics involved in potential hyperkalemia included spironolactone and amiloride

3 Comorbidities associated with the presence of potentially clinically significant DDIs

The following comorbidities were associated with the presence of potentially clinically significant DDI: chronic obstructive pulmonary disease (Odds ratio, OR = 5.1), asthma (OR = 2.2), atrial fibrillation (OR = 2.3), depression and/or anxiety (OR = 2.2), peripheral artery disease (OR = 2.0), heart failure (OR = 2.0), arterial hypertension (OR = 1.9), and chronic pain (OR = 1.5).

Supplementary Figure S1 shows the possible relationship between these comorbidities, indicated medication classes, and potential harms resulting from potentially clinically significant DDIs between these medication classes.

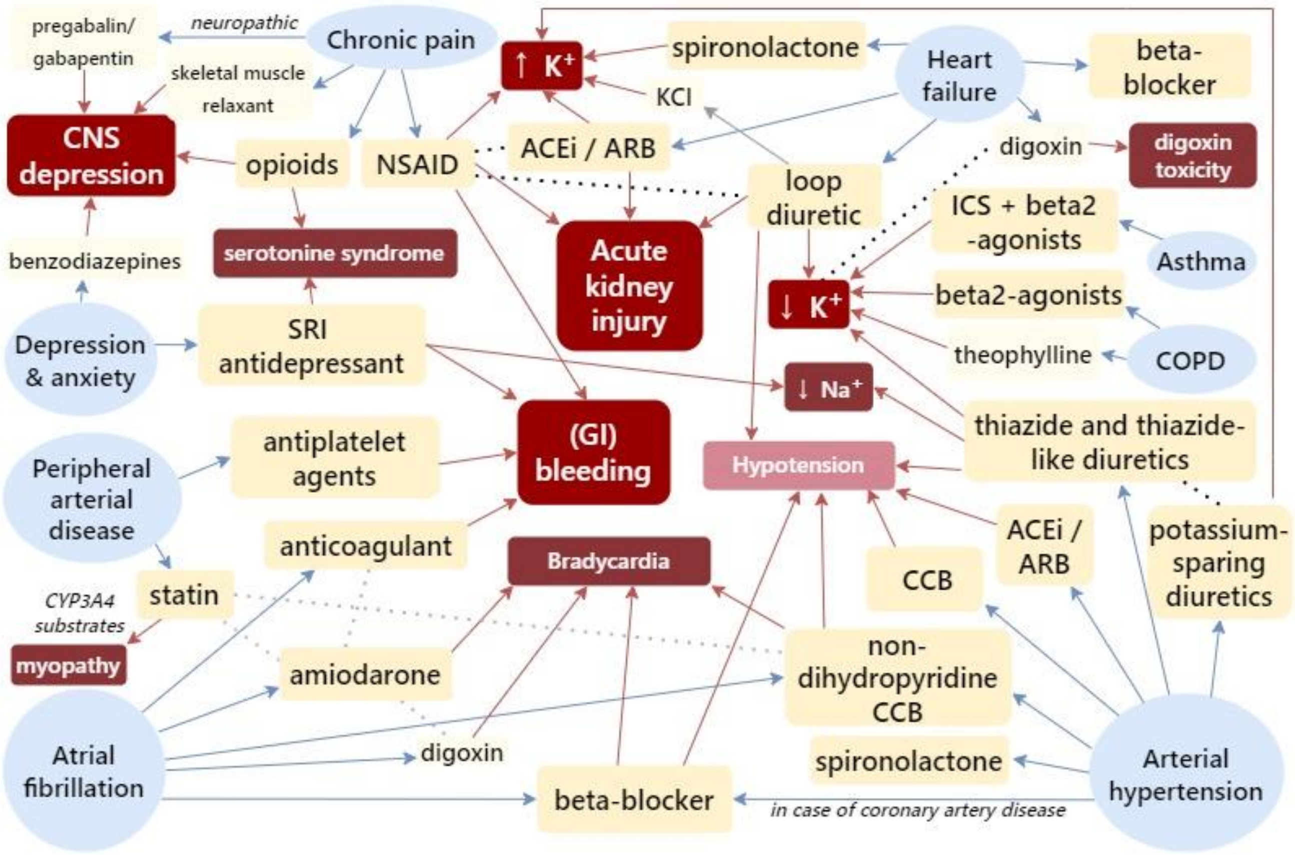

Supplementary Figure S1: Comorbidities associated with the presence of potentially clinically significant DDIs and their possible relationships with indicated medication classes and potential harms resulting from potentially clinically significant DDI between these medication classes.

ACEi: Angiotensin-converting enzyme inhibitors, ARB: Angiotensin receptor blockers, CCB: Calcium channel blocker, CNS: central nervous system, COPD: Chronic obstructive pulmonary disease, CYP: Cytochrome P450, GI: Gastrointestinal, ICS: Inhaled corticosteroids, NSAID: Non-steroidal anti-inflammatory drug, SRI: Serotonin reuptake inhibitor

Notes:

- *DDIs associated with hypotension were not listed in the consensus list.*
- *Only certain opioids might lead to serotonin syndrome (e.g., tramadol, tapentadol, fentanyl)*
